# Comprehensive management of epilepsy in onchocerciasis-endemic areas: lessons learnt from community-based surveys

**DOI:** 10.1186/s40249-019-0523-y

**Published:** 2019-02-10

**Authors:** Joseph Nelson Siewe Fodjo, Marieke C. J. Dekker, Richard Idro, Michel Ndahura Mandro, Pierre-Marie Preux, Alfred K. Njamnshi, Robert Colebunders

**Affiliations:** 1 Global Health Institute, University of Antwerp, Antwerp, Belgium; 2 Department of Internal Medicine and Pediatrics, Kilimanjaro Christian Medical Centre, Moshi, Tanzania; 3 Department of Neurology, Radboud University Medical Centre, Nijmegen, The Netherlands; 4 Department of Paediatrics and Child Health, Makerere University, College of Health Sciences, Kampala, Uganda; 5 Provincial Health Division Ituri, Ministry of Health, Bunia, Democratic Republic of the Congo; 6 INSERM, Univ. Limoges, Tropical Neuroepidemiology, Institute of Neuroepidemiology and Tropical Neurology, GEIST, 87000, Limoges, CHU, UMR 1094 Limoges, France; 7 Department of Neurology, Yaoundé Central Hospital / FMBS, The University of Yaoundé 1, Yaoundé, Cameroon; 8 Brain Research Africa Initiative (BRAIN), Yaoundé, Cameroon

**Keywords:** Epilepsy, Onchocerciasis, Community-based approach, Comprehensive management

## Abstract

**Background:**

Onchocerciasis*-*endemic regions are known to have a high epilepsy prevalence. Limited resources in these areas and poor access to healthcare by persons with epilepsy (PWE) result in a wide anti-epileptic treatment gap, poor seizure control and a high burden of seizure-related complications. Recent community-based surveys highlight the need for epilepsy management strategies suitable for remote onchocerciasis-endemic villages to ensure better health outcomes for PWE. In this paper, we propose a feasible approach to manage PWE in such settings.

**Main text:**

Improved management of PWE in onchocerciasis-endemic areas may be achieved by decentralizing epilepsy care. Simplified approaches for the diagnosis and treatment of epilepsy may be used by non-physicians, under the supervision of physicians or specialists. To reduce the treatment gap, a regular supply of subsidized anti-epileptic drugs (AED) appropriate for different types of onchocerciasis-associated epilepsy should be instituted. Setting up a community-based epilepsy surveillance system will enable early diagnosis and treatment of PWE thereby preventing complications. Community awareness programs on epilepsy must be implemented to reduce stigma and facilitate the social rehabilitation of PWE. Finally, strengthening onchocerciasis elimination programs by optimizing community-directed treatment with ivermectin (CDTI) and considering alternative treatment strategies might reduce the incidence of epilepsy.

**Conclusions:**

A community-based approach with task-shifting of epilepsy care from specialists to non-physician health workers will reduce epilepsy-associated morbidity. Increased advocacy and collaboration with various stakeholders is needed to establish a sustainable, cost-effective chronic care model for epilepsy that will significantly improve the quality of life of PWE in onchocerciasis-endemic regions.

**Electronic supplementary material:**

The online version of this article (10.1186/s40249-019-0523-y) contains supplementary material, which is available to authorized users.

## Multilingual abstracts

Please see Additional file 1 for translation of the abstract in the five official working languages of the United Nations.

## Background

**Fig. 1:**
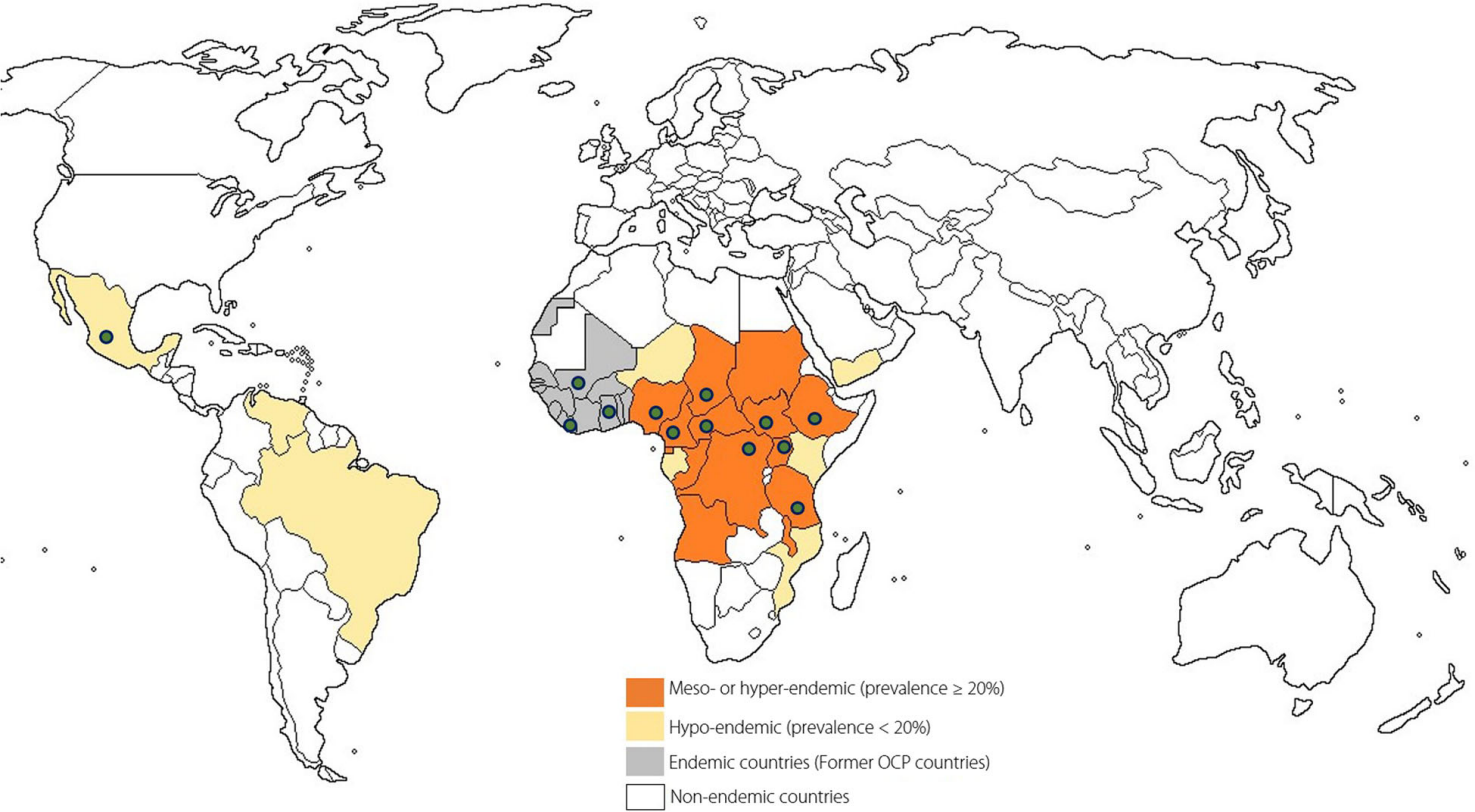
Map showing onchocerciasis endemic areas. The dots indicate countries where different forms of OAE have been reported [13, 18]

Epilepsy is highly prevalent in onchocerciasis*-*endemic regions, particularly in areas where the intensity of infection is high [1–3]. The relationship between onchocerciasis and epilepsy has further been underlined by two meta-analyses [4, 5] as well as recent community-based surveys in rural onchocerciasis-endemic villages, where 3.5–7.8% of the population have epilepsy [6–9]. Although onchocerciasis is classically known to only cause skin and eye disease, there is accumulating evidence suggesting that *Onchocerca volvulus* (the parasite that causes onchocerciasis), may directly or indirectly, be causing a wide spectrum of seizure disorders now described as onchocerciasis-associated epilepsy (OAE) [10, 11] (Fig. 1). Indeed, childhood infection with *O. volvulus* was shown to augment the risk of epilepsy in a microfilaria load-dependent manner [10]*.* In addition to generalized tonic-clonic seizures, absences and focal seizures, OAE also includes two clinical entities namely the nodding syndrome (involuntary, brief head nodding movements of sudden onset with reduced consciousness, possibly associated with declining cognitive function) and the Nakalanga syndrome (stunting, delayed or absent secondary sexual characteristics, deformities and/or epilepsy) [12–14]. An estimated 381 000 individuals currently suffer from OAE [15]; however the exact pathophysiological mechanism by which onchocerciasis may cause epilepsy is yet to be determined and treatment of affected persons remains sub-optimal.

Nearly 80% of persons with epilepsy (PWE) live in low- and middle-income countries [16] including sub-Saharan Africa, which also harbours more than 99% of cases of onchocerciasis [17, 18]. The epilepsy treatment gap in these regions is wide, with an estimated 75% of PWE not receiving adequate treatment [16]. This results in frequent epilepsy-related morbidity including a high burden of uncontrolled seizures, burns, traumatic injury and drowning [19]. Also, the epileptic seizures and the itching of onchodermatitis result in stigmatization of the affected individuals [19–21] thus increasing the psycho-social burden of OAE. Consequently, many PWE remain hidden from the public and undetected by healthcare services [22], leading to late diagnosis of the condition and frequent complications. Mortality amongst PWE living in onchocerciasis-endemic areas is 6.2 to 7.2 times higher than in the general population [23, 24], compared to only 2.6 times in non-endemic settings [25]. As the peak ages of onset of OAE lie between 3 and 18 years [11], many untreated youngsters suffer from recurrent seizures and progressive neurological deterioration, with negative socio-economic repercussions. Moreover, the affected younger generation becomes an additional care burden, being unable to cater for the older generation. Such a huge epilepsy burden necessitates an intervention tailored to meet the specific needs of these communities. The goal of this paper is to address the medical, psycho-social and economic burden faced by PWE and their families.

### Main text

We performed a selective search on PubMed for relevant articles focusing on epilepsy management in resource-limited settings, including onchocerciasis-endemic areas. We also took into account recommendations from the World Health Organization (WHO) [26] and conclusions emerging from multidisciplinary working group discussions during the first international workshop on OAE [27].

### Current epilepsy management and difficulties encountered

In remote onchocerciasis-endemic villages in Africa, access to healthcare and anti-epileptic drugs (AED) is generally difficult [11]. PWE have to walk for long distances to the health facility, only to be received by personnel not trained in epilepsy care [11]. Moreover, the health facilities seldom have AED in stock and even when they do, finances still constitute a major limitation to PWE receiving treatment [28]. Most often, there is no clear epilepsy management plan in these areas, and PWE must strive to obtain AED all by themselves. In the absence of community and political engagement in epilepsy care in these villages, many PWE and their families resort to traditional healers [28], thus further delaying medical treatment while the symptoms worsen [29]. The current strategy for epilepsy management fails to detect PWE in the community, does not narrow the treatment gap and produces poor health outcomes for PWE.

During our recent surveys in onchocerciasis-endemic villages [6–9], we noted some common factors which must be considered before designing an epilepsy intervention. These include: remoteness of the onchocerciasis*-*endemic villages, poor accessibility, general poverty, inadequate healthcare services (little or no health infrastructure and/or personnel, frequent medication shortages), high epilepsy prevalence, poor health seeking behaviour leading to late diagnosis and frequent complications (cognitive symptoms, psychiatric problems, burns, trauma due to falls, submersion injuries), low literacy rate with frequent school drop outs at the primary level, frequent sexual abuse, misconceptions and stigma [11, 19, 30].

### Proposed plan for comprehensive epilepsy management

It is expedient that PWE in onchocerciasis-endemic areas receive appropriate treatment and that new cases of OAE be prevented by strengthening onchocerciasis elimination in endemic areas [11, 14]. Epilepsy is a condition that necessitates a chronic disease management plan that is both comprehensive and sustainable. We propose a framework for decentralization of care to local epilepsy clinics with task shifting from neurologists to basic health personnel and community health workers. The use of community volunteers for epilepsy care has been shown to be feasible and cost-effective in sub-Saharan Africa [31] including onchocerciasis-endemic settings [32]. Procedures for epilepsy diagnosis and management must be simplified for easy application by non-physicians (nurses and clinical officers), with zonal supervision by physicians. A network of specialists should be readily available by telephone for advice or for referral of complex cases. The different aspects of a comprehensive community-based epilepsy program are discussed below (see Additional file 2).

#### Screening for epilepsy

We propose to use the following definition of epilepsy: “the occurrence of at least two unprovoked seizures separated by at least 24 hours” [33]. The term “unprovoked” means that common causes of acute seizure such as febrile illness, infection of the central nervous system and substance abuse are not incriminated. Of the numerous tools that have been developed for epilepsy screening [34–37], the most suitable for use by basic health personnel/community workers is the 5-questions format developed by the Institute of Neurological Epidemiology and Tropical Neurology of Limoges (France) [37]. This tool was specifically elaborated for Africa with the support of the Pan-African Association of Neurological Sciences and the International League Against Epilepsy (Commission on Tropical Diseases, 1993–1997). It has the advantages of being brief, usable by non-physicians and showed a high sensitivity of 95.1% as well as an acceptable specificity of 65.6% during its validation in Mauritania [37]. It is also effective in diagnosing seizure types other than generalized tonic-clonic episodes, such as absences and focal seizures. However, because of the low literacy levels in resource-poor settings, the questions need to be asked in ways the local population will easily understand (Table 1). The questions should be translated in the local languages and back-translated for validation prior to use, and those who will administer the tool must be trained to properly understand how to ask the questions.

**Table 1:** Adaptation of the epilepsy screening questions to the local context

Questions in scientific language [37]	Questions explained and adapted to the context	Remark
1. Loss of consciousness and/or micturition and/or drooling? *Sensitivity: 87.8%* *Specificity: 90.3%*	Does the person suddenly fall for a short period of time (few seconds to minutes)? During such falls, is there saliva (foam) on the mouth and/or urine on the victim?	- Sudden fall implies that the victim has no time to hold onto a support before falling. - Micturition and drooling are usually absent during syncope, dizziness or hysteria but typically present during generalized tonic-clonic seizures.
2. Absences or sudden lapse of consciousness for a short duration? *Sensitivity: 50.0%* *Specificity: 86.6%*	Does the person suddenly stop talking/eating/working for a short period of time (few seconds), and does not respond when you call him/her? After that, does she/he resume to what she/he was doing?	- Questions must relate to common activities such as farming, talking and eating because it is the easiest way to notice abnormal events. - If the PWE cannot recall the episodes of brief lapse of consciousness meanwhile it is reported by the caretakers, it is most likely an absence.
3. Jerky or uncontrolled movements of one or more limbs (convulsions), of sudden onset and lasting for a few minutes? *Sensitivity: 69.5%* *Specificity: 79.9%*	Does the person get sudden shaking of the whole body or just part of the body (hands, legs) and this then calms down after a short while?	Generalized tonic-clonic seizures are easily recognized and may have a local appellation. It could be helpful to ask the interviewee to mimic the abnormal movements. Ask also whether the person experiences isolated movements of the head (nodding seizures). Importantly, do not suggest answers.
4. Sudden onset of brief body sensations, hallucinations or illusions be they visual, auditory or olfactory? *Sensitivity: 37.8%* *Specificity: 80.5%*	While fully awake, does the person often complain of abnormal body sensations, or seeing/ hearing/ smelling things that are not really there for a short period (few seconds to minutes)?	Non-motor seizures are difficult to explain. The best approach is to create a scenario of visual or auditory hallucinations (seeing people who are not present, hearing voices). In some cases, the symptom is well known but had never been attributed to epilepsy. Use the local word for hallucinations when screening, if it exists.
5. Had it been said before that the subject had epilepsy or had presented epileptic seizures? *Sensitivity: 70.7%* *Specificity: 94.8%*	Ask the question using the local word for persons with epilepsy in that community.	Most families will readily answer this question. However, more tact is needed to pull out cases that are generally hidden by the family because of associated stigma [64].

#### Diagnosis of epilepsy

In case of a positive response to any of the 5 screening questions, the second step should be to refer the person to a trained nurse or clinical officer to confirm or reject the diagnosis of epilepsy using another questionnaire (Additional file 3, Appendix 1). This confirmatory questionnaire could be printed on paper or developed as a mobile telephone application [38] to ease information exchange with specialized health personnel in case the diagnosis needs to be further discussed. In confirmed PWE, the likelihood of OAE in the community can further be investigated using the following criteria [14]: (i) History of at least two unprovoked epileptic seizures 24 h apart; (ii) Person living for at least three years in an onchocerciasis*-*endemic region; (iii) A high prevalence of epilepsy in the village and families having more than one child with epilepsy; (iv) No other obvious cause for the epilepsy (mainly by good history taking to exclude common etiologies such as perinatal, infectious or traumatic causes); (v) Onset of epilepsy between the ages of 3 and 18 years; (vi) Normal psycho-motor development before the onset of epilepsy. Local health personnel working in onchocerciasis-endemic settings are encouraged to check for exposure to *O. volvulus* in PWE either clinically (presence of characteristic skin lesions such as leopard skin, onchodermatitis and/or nodules) and/or biologically (skin snips, Ov16 antibody testing using rapid diagnostic tests).

**Fig. 2:**
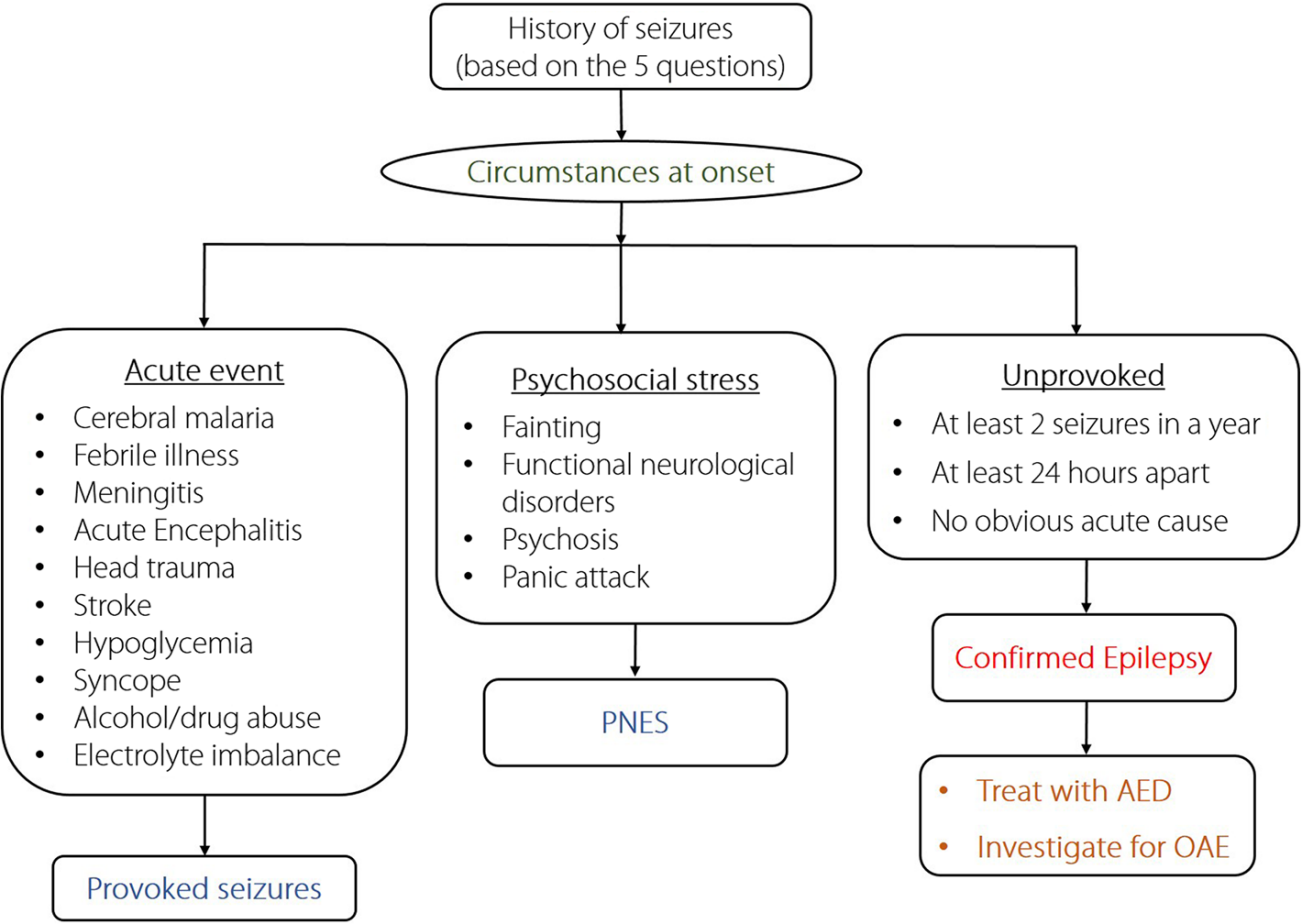
A simplified approach to the differential diagnoses of epilepsy

It is important to train the local health personnel on how to distinguish epilepsy from the following differential diagnoses: acute seizures (due to fever or an ongoing intracranial insult) [39], temporary loss of awareness (due to syncope or transient ischaemic attack), focal brain lesions (due to an abscess, hematoma or ischaemia), and psychiatric disorders including psychogenic non-epileptic seizures (PNES) [40] (Fig. 2). The training should be done by neurologists and/or physicians with specialized expertise in epilepsy management, who would continue to have a supervisory role in the management program [41].

#### Epilepsy treatment

##### Antiepileptic treatment

Epileptic seizures are treated using anti-epileptic drugs (AED) but only a limited number of AED are available and/or affordable in low resource settings [42]. AED that are routinely used in onchocerciasis-endemic regions include phenobarbital, carbamazepine, phenytoin, and valproate [7, 28, 32]. Their indications and prescribed dosages as recommended by the WHO [26] are detailed in Table 2. AED treatment must be initiated as monotherapy with progressive dose increase based on the response to the treatment and seizure control. Phenobarbital, the most available and affordable AED (annual cost per PWE: USD 5 [42]), is used as first line treatment for most seizure types but is not recommended for absences. It is usually initiated at 2–3 mg/kg/day and could be increased every 2–3 weeks by 15 mg if seizures continue, without going above the maximal recommended dose. When switching to another AED, phenobarbital should be tapered progressively (15 mg reduction every two weeks) while starting the newly prescribed AED as soon as the tapering begins. This approach minimizes the risk of rebound seizures upon stopping phenobarbital [43]. To ease administration of the right drug dosage, both phenobarbital 30 mg and 100 mg should be available. Switching to another drug is indicated if there is no decrease in seizure frequency using the right dosage of AED and/or when there are unacceptable side effects [43]. Polytherapy must be discussed with a specialist if two different drugs have failed [26]. Importantly, PWE must be educated that good seizure control can only be achieved by consistently taking AED every day and not just after a seizure.

**Table 2:** Description of common first-line AED in resource-limited settings

Drug	Indication and frequency of use	Required dosage		Remark
		Children	Adults	
Phenobarbital	Recommended by WHO as first line AED for most seizure types, except absences; cheap and readily available [19, 26]. Used by 74.6% of PWE [65].	Given once/twice daily Starting dose: 2–3 mg/kg/day Maintenance: 3–6 mg/kg/day	Given once daily Starting dose: 60 mg/day Maintenance: 60–180 mg/day	Steady state reached after 14–21 days. Possible side effects: Drowsiness, skin rash, lethargy and hyperactivity in children, hepatic failure, Stevens Johnson syndrome
Carbamazepine	Indicated for focal seizures, and could be used in generalized convulsive seizures [43]. Used by 27.4% of PWE [65]	Given twice daily Starting dose: 5 mg/kg/day Maintenance: 10–30 mg/kg/day	Given twice daily Starting dose: 100–200 mg/day Maintenance: 400–1400 mg/day	Steady state reached in 8 days. Possible side effects: allergic skin reactions, bone marrow suppression with long-term use, blurred vision, diplopia, ataxia, nausea. Contraindicated in absences and myoclonus [43]
Phenytoin	Indicated for treating some generalized seizures and status epilepticus [66]. Used by 22.2% of PWE [65]	Given once/twice daily Starting dose: 3–4 mg/kg/day Maintenance: 3–8 mg/kg/day (max 300 mg/day)	Given once or twice daily Starting dose: 150–200 mg/day Maintenance: 200–400 mg/day	Possible side effects: drowsiness, ataxia, slurred speech, motor twitching and mental confusion, coarsening of facial features, hepatitis, gum hyperplasia, hirsutism, skin reaction including Stevens Johnson syndrome
Valproate	A broad spectrum anticonvulsant that can be used for both focal and generalized onset seizures. Specifically indicated for absence, atonic and myoclonic seizures [66]. Preferred drug for nodding seizures [32]. Used by 14.7% of PWE [65]	Given twice daily Starting dose: 5–10 mg/kg/day Maintenance: 15–30 mg/kg/day	Given twice daily Starting dose: 400 mg/day Maintenance: 400–2000 mg/day	Possible side effects: sedation, tremor, transient hair loss, increase in body weight, impaired hepatic function. Use in women of childbearing age is discouraged

For PWE taking antiretroviral drugs (ARV), valproate is preferred as it presents less interactions with ARV [44]. There is a risk of foetal malformations with all the above mentioned AED, particularly during the first three months of gestation. If AED treatment is absolutely required during pregnancy, carbamazepine could be considered at the minimal effective dose, because it has the least risk of malformations and seems to have no negative effect on the neurodevelopment of the child later in life [45, 46]. Several studies have clearly demonstrated the teratogenicity of valproate and it should therefore be avoided in women of childbearing age and during pregnancy [46]. Moreover, unplanned pregnancies in female PWE should be discouraged and may be prevented by providing contraception. Barrier methods (condoms in particular) are most feasible as they are cheap, available and unaffected by AED unlike hormonal methods [47]. Supplementation with folic acid (4 mg/day) before and throughout pregnancy is encouraged as this reduces the risk of neural tube defects [48].

##### Additional therapies

For public health reasons and also as a specific treatment measure, PWE residing in onchocerciasis-endemic areas should receive ivermectin at least once a year [49] at the recommended dosage of 150 μg/kg alongside all other inhabitants of the endemic villages. Ivermectin is however mostly active against the microfilaria or larva, but exhibits only a partial effect on the adult *O. volvulus* or macrofilaria after repeated consecutive doses [50]. The once or twice annual treatment with ivermectin is needed for 15 years or more to achieve elimination [51]. Although current guidelines exclude pregnant women and children under five years from receiving ivermectin, we recommend that the safety of ivermectin should be investigated in children under five years [52] because the accumulation *O. volvulus* microfilaria in the absence of treatment increases the risk for OAE [10]. In persons with NS and Nakalanga syndrome, nutritional rehabilitation and additional specific measures may be required [32].

### Treatment and care of complications

PWE are at risk of seizure-related injuries and have more frequent accidents even without a seizure [53]. Common complications directly related to seizures include burns, traumatic lesions and submersion injuries. The ILAE has proposed guidelines to prevent seizure-related injury particularly in children [54]; these include close monitoring during bathing, sports, or when using open fires or the oven. Applying these guidelines in onchocerciasis-endemic settings would imply that PWE should not go to the well or river alone, nor go to the farm or sit near open cooking places without surveillance. Fire stoves should be made more secure and elevated to avoid PWE falling into them during a seizure. Furthermore, the bed of PWE should be as low as possible to prevent falls from a great height during a night seizure. Rooms of PWE should be void of unnecessary furniture and sharp objects which could hurt the PWE in the event of a seizure.

Burn injuries should urgently be cooled with clean running water for about 20 min, and then the victim should be rushed to a health facility. The health personnel should perform thorough debridement and adequately dress the wound. Local antiseptics could be applied. Tetanus immunization associated with serotherapy, and empirical antibiotic treatment should be given. As for submerged (drowning) PWE, they should rapidly be rescued from water and given cardiopulmonary resuscitation as required: repeated cycles of 30 chest compressions and two rounds of mouth-to-mouth ventilation [55]. Upon transportation to the health facility, they should be half-seated and any asthma-like breathing difficulties as a result of reflex bronchospasm can be relieved symptomatically using inhaled salbutamol [56]. Some epilepsy-related complications such as severe burns, severe traumatic injury, cognitive disabilities and psychiatric problems would require consultation with specialists via the telephone or having visiting specialists on a regular basis. If none of these is possible, there would be need for referral to a more specialised health facility.

#### Epilepsy clinic

Treatment for PWE should be dispensed through epilepsy clinics established at primary health care facilities, which should serve as the focal points to ensure epilepsy care and AED delivery. A senior nurse/clinical officer at the health facility should be trained to head the clinic; his/her duties will be to consult suspected cases of epilepsy, confirm the diagnosis, prescribe AED, evaluate for adverse effects and treatment outcomes and notify doctors/specialists in case of treatment failure, side-effects or complications. Nurses from target zones can be collectively trained during periodic seminars supervised by competent specialists and should be given practical manuals to ease implementation. Physicians trained in epilepsy care and/or specialized doctors working in nearby health structures are encouraged to regularly visit the clinics to personally consult some PWE and offer field supervision to the clinic heads.

Another task for the head of the clinic is to ensure the continuous supply of AED by ordering them every month based on the needs. Given the low purchasing power of the affected population, we propose a participatory approach for AED purchase whereby the affected families pay a yearly token (USD 2–USD 3 per PWE per year) which will be complemented by the government, local authority, or a partner organization to ensure an uninterrupted supply of AED all year round. This will have the double advantage of ensuring sustainability and increasing treatment adherence by PWE as they seek to fully utilize their invested funds. Epilepsy clinics should negotiate directly with accredited AED suppliers to deliver the drugs on site. These AED suppliers could benefit from certain privileges (such as tax reduction or exemption) to ensure continuous availability of routinely used AED at affordable costs. Valproate is much more expensive than the other AED, and should be subsidized to encourage treatment adherence. Indeed, we have observed that certain PWE switch from one AED to another or may stop treatment because the drugs are too expensive.

An important aspect of the clinic is proper documentation of all patient-related data and activities of the clinic. This will be done primarily using reporting forms adapted from the WHO training modules on mental health gap action program [57] for this purpose (see Additional file 3). The following parameters should be monitored monthly (or less often if seizure frequency is < 1 per month):

- Clinical features: anthropometric parameters, physical examination, seizure characteristics, complications, co-morbidities, deaths
- Treatment outcomes: adherence to AED, seizure frequency, side effects
- Quality of life: degree of autonomy, socialization, school/work, anxiety/depression
- Epilepsy-related expenses: cost of AED and care, transport cost to health centre, number of days of work lost
- Management: AED and dosage, other measures, date for next appointment

Epilepsy clinics should equally provide psycho-social support to PWE and their families. This can be achieved via support group sessions with patient-carer associations, during which advice and tips are given to PWE to equip them for healthier and more autonomous lives. Practical issues to be discussed include: treatment adherence, balanced diet, sufficient sleep (≥ 7 h per day), avoiding substance/alcohol abuse, physical exercise such as jogging, and school/work resumption. PWE should be encouraged to share their success stories so as to encourage others.

#### Community health workers (CHW)

CHW constitute the support staff and community arm of the epilepsy clinic. The clinic head should train them to suspect epilepsy using the 5-question approach and refer suspected PWE to the clinic for confirmation. As such, they will be instrumental in community-based surveillance of epilepsy by tracking suspected cases within their communities and referring them to the clinic for early diagnosis. Early diagnosis allows for epilepsy care to be initiated before the onset of complications. Also, a community-based surveillance system provides more realistic estimates of the burden of epilepsy, which is usually underestimated in hospital settings. It has been suggested that instead of CHW, community-directed distributors of ivermectin (CDD) could be trained for epilepsy screening, follow-up and surveillance in onchocerciasis-endemic areas [11].

CHW equally have to ensure community follow-up of PWE via home visits, ensuring good treatment adherence, monitoring treatment outcomes and reminding families of the monthly follow-up visits. They should monitor seizure frequencies of PWE and report in monthly forms (see Additional file 3, Appendix 3). For remote communities, CHW could collect AED from the clinic and deliver to the PWE in the village once a month. In addition, they are in charge of educating their respective communities about epilepsy in order to reduce stigma towards PWE. Some financial incentive may be required to boost the activities of CHW.

Traditional healers are highly sought after by affected families and should not be neglected when setting up an epilepsy management framework [19, 42]. Studies in Cameroon showed a willingness of traditional healers to collaborate with health professionals in epilepsy management [58]. Therefore, their role in the comprehensive management plan should be well defined [41]; after receiving basic education on epilepsy, they would be expected to educate families who bring PWE to them and refer suspected cases to the clinic. A modest compensation per PWE referred to the clinic and per family counselled could motivate traditional healers to actively participate in the management program.

Finally, it is necessary for the health personnel, CHW, traditional healers and the community at large to be sensitized about what to do when faced with an on-going seizure. Everyone should be aware of the following notions [26, 43]:

- Epilepsy is not transmissible like infectious diseases
- Do not panic or run away, abandoning the person
- Protect the person from injury: make sure they are in a safe place away from fire, water or other things that might injure them. Remove eye glasses, loosen tie or belt
- Do not hold the person tight in a bid to prevent the jerky movements
- Do not put anything into the mouth in an attempt to prevent tongue biting

Once the involuntary movements cease, put the person to lie on his/her side (recovery position) to prevent aspiration; check airway, breathing and pulse. A person with a prolonged seizure or short recurrent seizures within the same day should urgently be taken to the health centre.

#### Community awareness program and social rehabilitation of PWE

Outreach programs need to be organized in the community, schools, churches, markets and other public gatherings to sensitize the population about epilepsy. In onchocerciasis-endemic villages, clustering of PWE within some households fosters the wrong belief that epilepsy is transmissible by touch [59]. Primary care givers and the general public should be educated on the non-contagious nature of epilepsy and its non-mystical origin. Proper community education about epilepsy and the possible role of onchocerciasis in the clustering of PWE in some households will reduce epilepsy-related stigma and increase treatment adherence [59].

Epilepsy may affect education and other lifetime opportunities, income, social and professional fulfilment especially in low-income countries [60]. Younger PWE should be encouraged and assisted to go back to school (as most of them drop out at the onset of seizures). Parents and teachers should be informed that epilepsy does not necessarily render the PWE inept for schooling. Attractive policies such as free tuition, scholarships or other opportunities for PWE could galvanize them to effectively resume school. Furthermore, teachers should be trained on how to cater for the special needs of PWE who often have learning difficulties due to cognitive impairment [61]. This will entail adapting the academic curriculum for slow learners, exercising patience when teaching PWE, and training teachers to intervene efficiently in the event of a seizure. For the older PWE or those who cannot resume formal education for some reason, periodic capacity building training sessions should be organized to empower them with certain skills in agriculture, craftsmanship or other lucrative activities. More importantly, a sure market should be provided for their goods and services in order to sustain this strategy. This can best be achieved within the framework of patient-carer associations that PWE and their families are encouraged to form, to serve as support action and advocacy groups.

#### Strengthening onchocerciasis elimination programs

There is strong epidemiological evidence suggesting an association between epilepsy and onchocerciasis, with increasing onchocerciasis transmission resulting in increased epilepsy prevalence [3, 4, 10]. Therefore, strengthening onchocerciasis elimination programs is important to decrease the incidence of epilepsy [14]. In addition to frequent sensitization of the population in general and PWE in particular about the importance of taking ivermectin, advocacy is needed in favour of biannual ivermectin distribution in endemic communities. Where transmission remains very high, alternative treatment strategies such as river larviciding should be considered [62]. Similar interventions may have been partly or mainly associated with the prevention new cases of nodding syndrome in Uganda [63].

#### Sustainability of the epilepsy care program

**Fig. 3:**
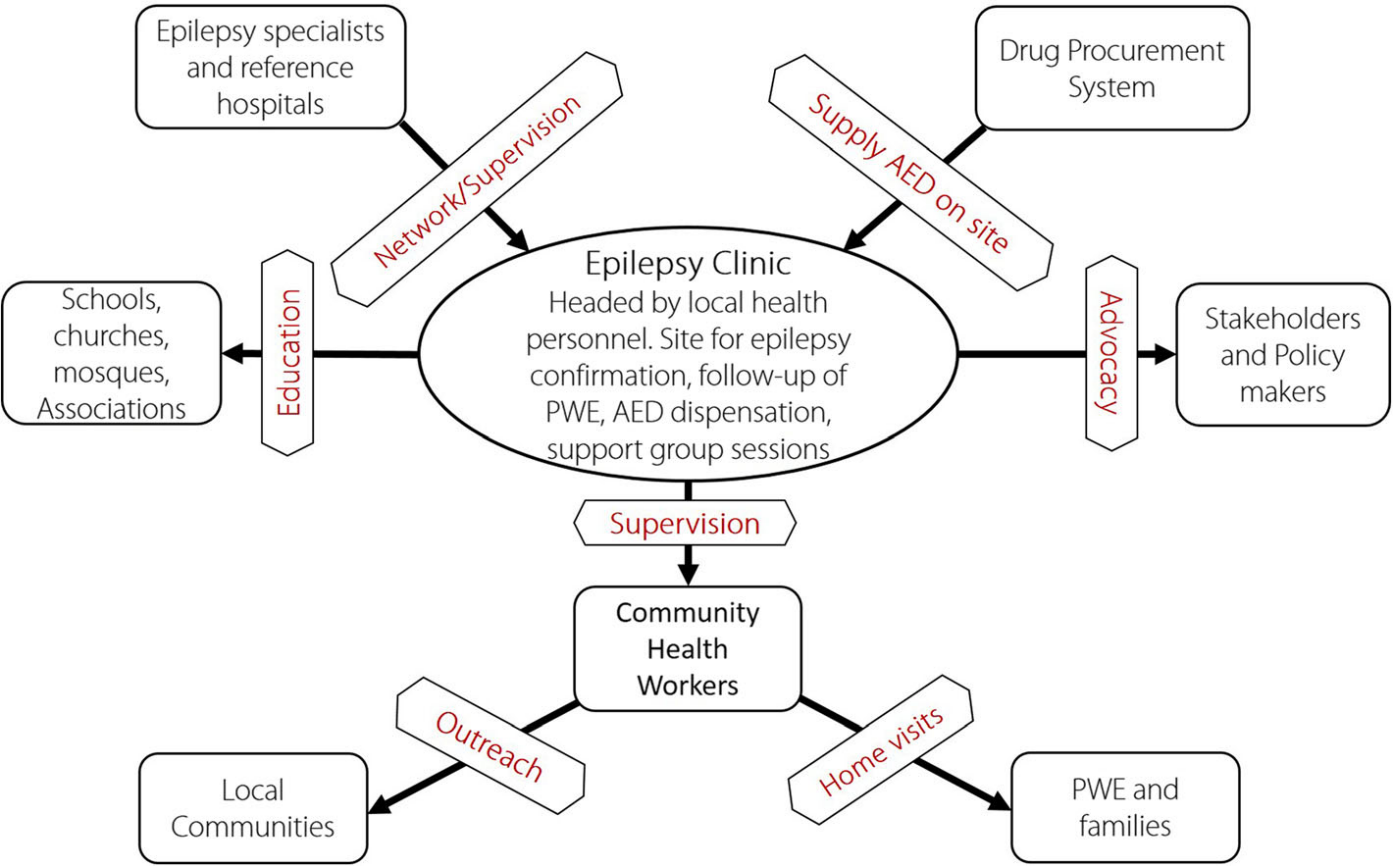
Relationship between different actors in the epilepsy program

To develop a sustainable comprehensive management program for PWE, a participative approach including integration into the national health system, must be established from the very start. A clear vision needs to be communicated to community leaders, elites, authorities, existing patient and advocacy associations to make them buy into the project early enough so as to participate in its implementation. Regular and concise reporting will improve credibility and build enough impact for the program to ensure sustainability. The local population should be convinced to consider epilepsy care as a priority community intervention in which they will voluntarily participate. Various parties in the epilepsy program and their roles are shown in Fig. 3. Some practical constraints that may arise when implementing the community-based epilepsy program and proposed mitigation strategies are summarized in Table 3.

**Table 3:** Constraints in implementing a community-based epilepsy program

Aspect	Possible constraint	Proposed solution
Decentralizing epilepsy care	Shortages in AED availability	Advocacy to prioritize onchocerciasis*-*endemic areas for AED delivery; work with multiple AED suppliers; advocacy with pharmaceutical firms to provide free/subsidized AED in endemic areas.
	Remote communities very far from health centres	Institute regular mobile clinics during which the nurse reaches out to remote communities. Can be coupled with other public health activities such as immunization, and maternal & child health services. AED could be transported monthly from the clinic to the village by the CHW.
Community awareness programs	Resistance by certain institutions (schools, churches, jobsites) to provide a platform for sensitization	Education of stakeholders about the importance of epilepsy sensitization in their respective institutions
	Difficulty in conveying the message in a contextual and convincing manner	Qualitative research could identify root problems and how to best address them
Evaluation and monitoring	Lack of qualified persons	Use simple reporting forms (see Additional file 3); Train more volunteers into CHW
	Epilepsy-related events not reported in some health systems.	Propose an epilepsy reporting form to be used in health structures in onchocerciasis*-*endemic areas
Strengthening onchocerciasis elimination programs	Insufficient public funds for bi-annual CDTI or alternative strategies	Advocacy to stakeholders about the importance of onchocerciasis elimination; make use of unpaid village volunteers for CDTI.
	Sub-optimal ivermectin intake by the population	Sensitization of the population about the importance of ivermectin to prevent epilepsy; better timing of distribution campaigns (avoid periods of intensive farming activity with high probability of meeting empty houses during distribution)
Sustainability of the program	Possibility of the program being closed after sometime due to lack of interest and/or resources	- Advocacy for stakeholders to include epilepsy programs among priority interventions in onchocerciasis-endemic regions - Integrate the national onchocerciasis and epilepsy programs to share resources and reduces waste
	Administrative bottlenecks and corruption	- Involve high ranking local elites who value the village and the populations

*CDTI* Community-directed treatment with ivermectin, *AED* Anti-epileptic drugs, *CHW* Community health workers

### Directions for further research

Knowledge gaps still exist in the domain of prevention and management of epilepsy in onchocerciasis*-*endemic regions. Performing research in these regions however is difficult because of their remoteness, the lack of medical and research infrastructure, lack of trained healthcare workers and local researchers. A few research priorities that should be addressed to ameliorate epilepsy care in these regions are outlined in Table 4.

**Table 4:** Research priorities on epilepsy management in onchocerciasis-endemic regions

Aspect	Research question	Possible research direction
Epilepsy screening	What is the optimal screening tool to detect PWE by non-physicians?	- Need to validate screening questionnaires in different settings - Evaluate the feasibility of using video-taped seizures as a diagnostic tool
Diagnosis of epilepsy	What is the risk of seizure recurrence in an *O. volvulus*-infected person after a first seizure?	Prospective study to follow up healthy children in highly endemic onchocerciasis settings. If recurrence risk is found to be ≥60%, then one seizure in the presence of onchocerciasis would be considered as epilepsy [33]
	How can OAE be predicted or diagnosed in an early phase?	Prospective study in onchocerciasis-exposed children with regular clinical, electrophysiological and brain imaging assessment; evaluation of biomarkers in blood, CSF, and/or skin snips
Epilepsy treatment	What is the optimal training module for first line healthcare workers to care for PWE?	Intervention study to test different training modules for epilepsy care
	What is the optimal anti-epileptic treatment for each type of OAE, including absences and nodding seizures?	Multi-center cohort study and if possible clinical trials to evaluate the efficacy and safety of different treatment regimens
	Is anti-onchocerciasis treatment able to reduce the frequency of seizures in persons with OAE?	Clinical trials to assess the effect of ivermectin [67] and doxycycline [68] on seizures are ongoing
Strengthening onchocerciasis elimination programs	Is it safe to give ivermectin to children < 5 years with onchocerciasis and possibly OAE?	- Clinical trials to obtain safety data about ivermectin use in children < 5 years [52] - Development of safer drugs for onchocerciasis which can be used in children < 5 years
Sustainability of the epilepsy care program	What is the impact and cost-effectiveness of a community-based epilepsy program?	Impact and cost analysis studies before and after the implementation of an epilepsy care program [31]. The following outcome indicators should be assessed: % PWE treated, % PWE adherent to treatment and seizure free, change in epilepsy-related mortality, % epilepsy complications, % PWE continuing education or working, change in direct cost of care per PWE, indirect cost averted by the epilepsy program, cost of epilepsy for the PWE, family and community; cost of running the epilepsy clinic/program

*CSF* Cerebrospinal fluid, *PWE* Person with epilepsy

## Conclusion

Epilepsy remains a major neglected public health problem in many onchocerciasis*-*endemic regions with sub-optimal control measures. Compared with HIV, another chronic stigmatizing condition, there is a large gap in treatment and care services available for PWE. In these regions, epilepsy stigma is further compounded by onchocerciasis stigma, thus limiting treatment-seeking behaviour. Establishing a comprehensive, decentralized community-based epilepsy treatment and care program that will equip non-physicians in the remote villages may be the way forward to improving epilepsy care. This requires proper advocacy and collaboration among many stakeholders – including pharmaceutical companies to provide cheap/free AED – and a strong political commitment for its implementation. Finally, onchocerciasis elimination programs should be reinforced to prevent future cases of OAE.

## Additional files

Additional file 1: Multilingual abstract in the five official working languages of the United Nations. (PDF 487 kb)

Additional file 2: Main components of a community-based epilepsy program. (DOCX 14 kb)

Additional file 3: Forms for epilepsy diagnosis and follow-up. (DOCX 80 kb)

## Abbreviations

AED: Anti-epileptic drug(s)
CDD: Community-directed distributors of ivermectin
CDTI: Community-directed treatment with ivermectin
CHW: Community health worker(s)
ILAE: International League Against Epilepsy
OAE: Onchocerciasis-associated epilepsy
PNES: Psychogenic non-epileptic seizures
PWE: Person(s) with epilepsy
USD: American dollar
WHO: World Health Organization

## References

[1] Kaiser C, Kipp W, Asaba G, Mugisa C, Kabagambe G, Rating D (1996). The prevalence of epilepsy follows the distribution of onchocerciasis in a west Ugandan focus. Bull World Health Organ.

[2] Dozie INS, Onwuliri COE, Nwoke BEB, Chukwuocha UM, Chikwendu CI, Okoro I (2006). Onchocerciasis and epilepsy in parts of the Imo river basin, Nigeria: a preliminary report. Public Health.

[3] Boussinesq M, Pion SD, Ngangue D, Kamgno J (2002). Relationship between onchocerciasis and epilepsy: a matched case-control study in the Mbam Valley, Republic of Cameroon. Trans R Soc Trop Med Hyg.

[4] Pion SD, Kaiser C, Boutros-Toni F, Cournil A, Taylor MM, Meredith SEO, et al. Epilepsy in onchocerciasis endemic areas: systematic review and meta-analysis of population-based surveys. PLoS Negl Trop Dis. 2009. 10.1371/journal.pntd.0000461. 10.1371/journal.pntd.0000461 PMC2691484 19529767

[5] Kaiser C, Pion SD, Boussinesq M. Case-control studies on the relationship between onchocerciasis and epilepsy: systematic review and meta-analysis. PLoS Negl Trop Dis. 2013. 10.1371/journal.pntd.0002147. 10.1371/journal.pntd.0002147 PMC3610636 23556028

[6] Mmbando BP, Suykerbuyk P, Mnacho M, Kakorozya A, Matuja W, Hendy A, et al. High prevalence of epilepsy in two rural onchocerciasis endemic villages in the Mahenge area, Tanzania, after 20 years of community directed treatment with ivermectin. Infect Dis Poverty. 2018. 10.1186/s40249-018-0450-3. 10.1186/s40249-018-0450-3 PMC6009039 29921319

[7] Lenaerts E, Mandro M, Mukendi D, Suykerbuyk P, Dolo H, Wonya’Rossi D, et al. High prevalence of epilepsy in onchocerciasis endemic health areas in Democratic Republic of the Congo. Infect Dis Poverty. 2018. 10.1186/s40249-018-0452-1. 10.1186/s40249-018-0452-1 PMC6069757 30064504

[8] Siewe FJN, Tatah GY, Tabah EN, Ngarka L, Nfor LN, Chokote SE, et al. Epidemiology of onchocerciasis-associated epilepsy in the Mbam and Sanaga river valleys of Cameroon: Impact of more than 13 years of ivermectin. Infect Dis Poverty. 2018. 10.1186/s40249%2D018%2D0497-1. 10.1186/s40249-018-0497-1 PMC6276171 30501640

[9] Colebunders R, Abd-Elfarag G, Carter JY, Olore PC, Puok K, Menon S, et al. Clinical characteristics of onchocerciasis-associated epilepsy in villages in Maridi County, Republic of South Sudan. Seizure. 2018; Accessed 6 Oct 2018]; Available from: https://linkinghub.elsevier.com/retrieve/pii/S1059131118306204. 10.1016/j.seizure.2018.10.004 30340162

[10] Chesnais CB, Nana-Djeunga HC, Njamnshi AK, Lenou-Nanga CG, Boullé C, Bissek A-CZ-K (2018). The temporal relationship between onchocerciasis and epilepsy: a population-based cohort study. Lancet Infect Dis.

[11] Colebunders R, Alfred K, Njamnshi OMV, Mukendi D, Kashama JM, Mandro M (2017). onchocerciasis-associated epilepsy: from recent epidemiological and clinical findings to policy implications. Epilepsia Open..

[12] World Health Organization. International Scientific meeting on Nodding Syndrome. Kampala, Uganda; 2012. Available from: https://www.who.int/neglected_diseases/diseases/Nodding_syndrom_Kampala_Report_2012.pdf?ua=1. Accessed 6 Mar 2018.

[13] Föger K, Gora-Stahlberg G, Sejvar J, Ovuga E, Jilek-Aall L, Schmutzhard E, et al. Nakalanga syndrome: clinical characteristics, potential causes, and its relationship with recently described nodding syndrome. PLoS Negl Trop Dis. 2017. 10.1371/journal.pntd.0005201. 10.1371/journal.pntd.0005201 PMC5300103 28182652

[14] Colebunders R, Nelson Siewe FJ, Hotterbeekx A (2018). Onchocerciasis-associated epilepsy, an additional reason for strengthening onchocerciasis elimination programs. Trends Parasitol.

[15] Vinkeles Melchers NVS, Mollenkopf S, Colebunders R, Edlinger M, Coffeng LE, Irani J, et al. Burden of onchocerciasis-associated epilepsy: first estimates and research priorities. Infect Dis Poverty. 2018. 10.1186/s40249-018-0481-9. 10.1186/s40249-018-0481-9 PMC6156959 30253788

[16] World Health Organization (2018). Epilepsy Fact sheet.

[17] World Health Organization (2017). Onchocerciasis Fact sheet.

[18] World Health Organization (2014). Distribution of Onchocerciasis worldwide, 2013.

[19] World Health Organisation (2004). Epilepsy in the WHO African region: bridging the gap.

[20] Wolf R, Orion E, Matz H (2003). Onchocerciasis (river blindness). Isr Med Assoc J.

[21] Tabah EN, Yepnjio F, Njamnshi AK, Bentivoglio M, Cavalheiro EA, Kristensson K, Patel NB (2014). Stigma in Neurological Diseases in the Tropics. Neglected Trop Dis Cond Nerv Syst.

[22] Meyer A-C, Dua T, Ma J, Saxena S, Birbeck G (2010). Global disparities in the epilepsy treatment gap: a systematic review. Bull World Health Organ.

[23] Kamgno J, Pion SDS, Boussinesq M (2003). Demographic impact of epilepsy in Africa: results of a 10-year cohort study in a rural area of Cameroon. Epilepsia.

[24] Kaiser C, Asaba G, Kasoro S, Rubaale T, Kabagambe G, Mbabazi M (2007). Mortality from epilepsy in an onchocerciasis-endemic area in West Uganda. Trans R Soc Trop Med Hyg.

[25] Wagner RG, Bottomley C, Ngugi AK, Ibinda F, Gómez-Olivé FX, Kahn K, et al. Incidence, remission and mortality of convulsive epilepsy in rural Northeast South Africa. PLoS One. 2015. 10.1371/journal.pone.0129097. 10.1371/journal.pone.0129097 PMC4459982 26053071

[26] World Health Organization (2010). mhGAP intervention guide for mental, neurological and substance use disorders in non-specialized health settings: version 1.0.

[27] Colebunders R, Mandro M, Njamnshi AK, Boussinesq M, Hotterbeekx A, Kamgno J, et al. Report of the first international workshop on onchocerciasis-associated epilepsy. Infect Dis Poverty. 2018. 10.1186/s40249-018-0400-0. 10.1186/s40249-018-0400-0 PMC5868050 29580280

[28] Dongmo L, Echouffo TJB, Njamnshi A, Poyi MK, Victor S, Nourdi PM. Difficulties faced in the management of epilepsy in rural Cameroon: the case of Mbangassina locality. Afr J Neurol Sci. 2003; Accessed 27 Feb 2018]. Available from: http://www.ajol.info/index.php/ajns/article/view/7533.

[29] Ma TM, Ma TM, Lelo GM, Nkosi MM, Madinga J, Kola CK, et al. Are the children with epilepsy treated traditionally a disadvantaged group? A pilot study. Pan Afr Med J. 2016. 10.11604/pamj.2016.23.229.9165.

[30] Wilmshurst JM, Kakooza-Mwesige A, Newton CR (2014). The challenges of managing children with epilepsy in Africa. Semin Pediatr Neurol.

[31] Van Diessen E, van der Maas F, Cabral V, Otte WM (2018). Community-based rehabilitation offers cost-effective epilepsy treatment in rural Guinea-Bissau. Epilepsy Behav.

[32] Idro R, Namusoke H, Abbo C, Mutamba BB, Kakooza-Mwesige A, Opoka RO, et al. Patients with nodding syndrome in Uganda improve with symptomatic treatment: a cross-sectional study. BMJ Open. 2014. 10.1136/bmjopen-2014-006476. 10.1136/bmjopen-2014-006476 PMC4244396 25398677

[33] Fisher RS, Acevedo C, Arzimanoglou A, Bogacz A, Cross JH, Elger CE (2014). ILAE official report: a practical clinical definition of epilepsy. Epilepsia.

[34] Konanki R, Mishra D, Gulati S, Aneja S, Deshmukh V, Silberberg D (2014). INCLEN diagnostic tool for epilepsy (INDT-EPI) for primary care physicians: development and validation. Indian Paediatr.

[35] Ngugi AK, Bottomley C, Chengo E, Kombe MZ, Kazungu M, Bauni E, et al. The validation of a three-stage screening methodology for detecting active convulsive epilepsy in population-based studies in health and demographic surveillance systems. Emerg Themes Epidemiol. 2012. 10.1186/1742-7622-9-8. 10.1186/1742-7622-9-8 PMC3549939 23171721

[36] Giuliano L, Cicero CE, Crespo Gómez EB, Padilla S, Bruno E, Camargo M, et al. A screening questionnaire for convulsive seizures: A three-stage field-validation in rural Bolivia. Romigi A, editor. PLOS ONE. 2017; doi: 10.1371/journal.pone.0173945. 10.1371/journal.pone.0173945 PMC5354446 28301557

[37] Diagana M, Preux PM, Tuillas M, Ould Hamady A, Druet-Cabanac M (2006). Dépistage de l’épilepsie en zones tropicales: validation d’un questionnaire en Mauritanie. Bull Soc Pathol Exot.

[38] Patterson V. Telemedicine for epilepsy support in resource-poor settings. Front Public Health. 2014. 10.3389/fpubh.2014.00120. 10.3389/fpubh.2014.00120 PMC4139740 25191650

[39] Beghi E, Carpio A, Forsgren L, Hesdorffer DC, Malmgren K, Sander JW (2010). Recommendation for a definition of acute symptomatic seizure. Epilepsia.

[40] Dekker MCJ, Urasa SJ, Kellogg M, Howlett WP (2018). Psychogenic non-epileptic seizures among patients with functional neurological disorder: a case series from a Tanzanian referral hospital and literature review. Epilepsia Open..

[41] Njamnshi AK (2009). Nonphysician management of epilepsy in resource-limited contexts: roles and responsibilities. Epilepsia.

[42] Caraballo R, Fejerman N (2015). Management of epilepsy in resource-limited settings. Epileptic Disord.

[43] Howlett W (2012). Neurology in Africa: clinical skills and neurological disorders.

[44] Siddiqi O, Birbeck GL (2013). Safe treatment of seizures in the setting of HIV/AIDS. Curr Treat Options Neurol.

[45] Pennell PB (2008). Antiepileptic drugs during pregnancy: what is known and which AEDs seem to be safest?. Epilepsia.

[46] Weston J, Bromley R, Jackson CF, Adab N, Clayton-Smith J, Greenhalgh J, et al. Monotherapy treatment of epilepsy in pregnancy: congenital malformation outcomes in the child. Cochrane Epilepsy Group, editor. Cochrane Database Syst Rev. 2016 [Accessed 2018 Feb 13]; Available from: http://doi.wiley.com/10.1002/14651858.CD010224.pub2 10.1002/14651858.CD010224.pub2 PMC6465055 27819746

[47] Gerard EE, Meador KJ (2016). Managing epilepsy in women. Contin Lifelong Learn Neurol.

[48] Shorvon S (2002). Antiepileptic drug therapy during pregnancy: the neurologist’s perspective. J Med Genet.

[49] Frempong KK, Walker M, Cheke RA, Tetevi EJ, Gyan ET, Owusu EO (2016). Does increasing treatment frequency address suboptimal responses to ivermectin for the control and elimination of river blindness?. Clin Infect Dis.

[50] Walker M, Pion SDS, Fang H, Gardon J, Kamgno J, Basáñez M-G (2017). Macrofilaricidal efficacy of repeated doses of ivermectin for the treatment of river blindness. Clin Infect Dis.

[51] Traore MO, Sarr MD, Badji A, Bissan Y, Diawara L, Doumbia K, et al. Proof-of-principle of onchocerciasis elimination with ivermectin treatment in endemic foci in Africa: final results of a study in Mali and Senegal. PLoS Negl Trop Dis. 2012. 10.1371/journal.pntd.0001825. 10.1371/journal.pntd.0001825 PMC3441490 23029586

[52] Chosidow A, Gendrel D (2016). Tolérance de l’ivermectine orale chez l’enfant. Arch Pédiatrie.

[53] Van den Broek M, Beghi E (2004). RESt-1 group. Accidents in patients with epilepsy: types, circumstances, and complications: a European cohort study. Epilepsia.

[54] ILAE Commission report. Restrictions for children with epilepsy. Commission of Pediatrics of the ILAE. International League Against Epilepsy. Epilepsia. 1997;38:1054–6. 10.1111/j.1528-1157.1997.tb01493.x 9579949

[55] Hazinski MF, Nolan JP, Aickin R, Bhanji F, Billi JE, Callaway CW (2015). Part 1: executive summary: 2015 international consensus on cardiopulmonary resuscitation and emergency cardiovascular care science with treatment recommendations. Circulation.

[56] McCall JD, Sternard BT (2018). Drowning. StatPearls [internet].

[57] World Health Organization. WHO Mental Health Gap Action Programme. [Accessed 12 Dec 2017]. Available from: http://www.who.int/mental_health/mhgap/en/.

[58] Njamnshi AK, Bissek A-CZ-K, Yepnjio FN, Tabah EN, Angwafor SA, Kuate CT (2010). A community survey of knowledge, perceptions, and practice with respect to epilepsy among traditional healers in the Batibo Health District, Cameroon. Epilepsy Behav.

[59] Nelson SFJ, Robert C. Intervention for reducing epilepsy-associated stigma. Epilepsy Behav. 2018; Accessed 6 Oct 2018]; Available from: https://linkinghub.elsevier.com/retrieve/pii/S1525505018307157. 10.1016/j.yebeh.2018.09.011 30282593

[60] Hills MD (2007). The psychological and social impact of epilepsy. Neurol Asia.

[61] Duggan M. Epilepsy and its effects on children and families in rural Uganda. Afr Health Sci. 2013;13. 10.4314/ahs.v13i3.14 PMC3824457 24250298

[62] Boussinesq M, Fobi G, Kuesel AC (2018). Alternative treatment strategies to accelerate the elimination of onchocerciasis. Int Health.

[63] Colebunders R, Irani J, Post R (2016). Nodding syndrome—we can now prevent it. Int J Infect Dis.

[64] Nabi AR, Nasrabadi AN, Navab E (2017). Family stigma associated with epilepsy: a qualitative study. J Caring Sci.

[65] Ibinda F, Odermatt P, Kariuki SM, Kakooza-Mwesige A, Wagner RG, Owusu-Agyei S (2017). Magnitude and factors associated with nonadherence to antiepileptic drug treatment in Africa: a cross-sectional multisite study. Epilepsia Open.

[66] NICE (2012). Epilepsies: diagnosis and management [internet]. National Institute for health and care excellence.

[67] Colebunders R, Mandro M, Mukendi D, Dolo H, Suykerbuyk P, Van Oijen M. Ivermectin treatment in patients with onchocerciasis-associated epilepsy: protocol of a randomized clinical trial. JMIR Res Protoc. 2017. 10.2196/resprot.7186. 10.2196/resprot.7186 PMC5597797 28855148

[68] Anguzu R, Akun PR, Ogwang R, Shour AR, Sekibira R, Ningwa A, et al. Setting up a clinical trial for a novel disease: a case study of the doxycycline for the treatment of nodding syndrome trial – challenges, enablers and lessons learned. Glob Health Action. 2018. 10.1080/16549716.2018.1431362. 10.1080/16549716.2018.1431362 PMC5795749 29382251

