# Supplementary material for: Comprehensive management of epilepsy in onchocerciasis-endemic areas: lessons learnt from community-based surveys
Source: Infect Dis Poverty. 2019 Feb 10;8:11. doi: 10.1186/s40249-019-0523-y (PMC6368958; doi:10.1186/s40249-019-0523-y)
Supplement: Supplementary file 1 — Multilingual abstract in the five official working languages of the United Nations. (PDF 487 kb) [file 40249_2019_523_MOESM1_ESM.pdf]

Translation of the abstract into the five official working languages of the United Nations

العلاج الشامل لداء الصرع في المناطق التي ينتشر فيها مرض العمى النهري (الأنكوسركية أو داء كلابية الذنب): دروس مستخلصة من الاستقصاءات القائمة على المجتمع المحلي

جوزيف نيلسون سيو فودجو ، وماريك سي جيه ديكر ، وريتشارد إيدرو ، وميشيل ماندرو ندهورا ، وبيير ماري برو ، وألفريد ك. نجامنشي وروبرت كولبندرز

ملخّص

خلفيّة: تشتهر المناطق التي ينتشر فيها مرض العمى النهري بتفشي داء الصرع فيها انّ الموارد المحدودة في هذه المناطق وصعوبة حصول الأشخاص المصابين بداء الصرع على الرعاية الصحيّة عوامل تؤدي إلى اتساع الفجوة في علاج داء الصرع وسوء مراقبة النوبات المرضية والعبء الثقيل بسبب مضاعفات النوبات المرضية. ان الاستقصاءات القائمة على المجتمع المحلي سلّطت الضوء على ضرورة وجود استراتيجيات لمعالجة داء الصرع حيث تكون ملائمة للقرى النائية التي ينتشر فيها مرض العمى النهري من أجل ضمان نتائج صحيّة أفضل لمرضى الصرع.

النصّ الأساس: انّ تحسين العلاج لمرضى الصرع في المناطق التي ينتشر فيها مرض العمى النهري قد يتحقق من خلال العناية اللامركزية لداء الصرع. كما أنّ النهج المبسّطة لتشخيص وعلاج داء الصرع يمكن أن يستخدمها أشخاص غير أطبّاء تحت اشراف أطبّاء أو متخصّصين. للحدّ من فجوة العلاج، ينبغي تقديم إمدادات منتظمة منالأدوية المدعومة لعلاج الصرع والأدوية المناسبة لمختلف أنواع الصرع الناتج عن مرض العمى النهري. انّ وضع نظام مراقبة قائمة على المجتمع المحلي لداء الصرع سيسنح التشخيص والعلاج المبكرين ممّا يؤدّي الى الوقاية من المضاعفات. ويتعين تنفيذ برامج التوعية المجتمعية لداء الصّرع للحدّ من آثار الوصم وتسهيل إعادة التأهيل الاجتماعي لمرضى الصّرع. وأخيرا، إنّ تعزيز برامج القضاء على مرض العمى النهري من خلال تحسين طرق علاج يتولى توجيهها المجتمع المحلي بايفيرمكتين ودراسة استراتيجيات العلاجات البديلة يمكن أنّ تحدّ من حالات الصّرع.

الخاتمة: الخاتمة: انّ النهج المجتمعي مع تحويل مهام العناية بداء الصرع من أخصائيين الى الموظفين الصحيّين غير الأطباء سيحدّ من معدّل الوفيّات المرتبط بداء الصّرع كما كما أنّ زيادة أنشطة الدعوة والتعاون مع مختلف الجهات المعنيّة يعتبر أمراً ضرورياً لإنشاء نموذج رعاية مستدام ومتواصل وفعّال من حيث التكلفة ضد داء الصّرع مما يحسّن بشكل ملحوظ من نوعية حياة مرضى الصرع في المناطق التي ينتشر فيها مرض العمى النهري.

Translated from English version into Arabic by Rana Chehab, through

TRANSLATORS
WITHOUT BORDERS

盘尾丝虫病流行区基于社区调查的癫痫综合管理经验

Joseph Nelson Siewe Fodjo, Marieke C. J. Dekker, Richard Idro, Michel Mandro Ndahura, Pierre-Marie Preux, Alfred K. Njamnshi and Robert Colebunders

摘要

引言： 盘尾丝虫病流行区的癫痫患病率较高。由于该类地区由于资源有限，癫痫患者无法得到有效医护和抗癫痫治疗，从而导致癫痫控制不佳以及相关并发症的高负担。当前的社区调查建议加强适应于偏远盘尾丝虫病流行区的癫痫管理策略，更好地保障癫痫患者的健康。

正文：在盘尾丝虫病流行区，分散照护可提高癫痫患者的管理，由非内科医生的卫生工作者

在内科医生或内科专业人员监督下操作简化的癫痫癫痫诊断和治疗措施。为更好实现癫痫治疗，建议成立适应各种盘尾丝虫病相关癫痫的补助抗癫痫药物常规供应；建立一个基于社区的癫痫监测系统，实现癫痫早诊断和早治疗，从而预防并发症；提高人群对癫痫知晓水平，降低人们对癫痫的误解，使患者更好地融入社会。最后，采用基于社区的伊维菌素治疗加强盘尾丝虫病消除项目，并寻找其他治疗方案降低癫痫的发病。
结论: 由非内科医生的卫生工作者接替专业的内科医生进行癫痫患者照护的社区措施将降低癫痫发病率。有必要通过强化多方的沟通与合作构建一种可持续、经济且有效的癫痫照护模式，从而显著提高盘尾丝虫病流行区癫痫患者的生命质量。

Translated from English version into Chinese by Cong-Shan Liu, edited by Jin Chen, through
CHINESE CENTER FOR DISEASE CONTROL AND PREVENTION
NATIONAL INSTITUTE OF PARASITIC DISEASES

Gestion globale de l’épilepsie dans les zones d’onchocercose endémique : enseignements tirés d’enquêtes communautaires

Joseph Nelson Siewe Fodjo, Marieke C. J. Dekker, Richard Idro, Michel Mandro Ndahura, Pierre-Marie Preux, Alfred K. Njamnshi et Robert Colebunders

Résumé
Contexte : On sait que la prévalence de l’épilepsie est élevée dans les régions d’onchocercose endémique. À cause des ressources limitées de ces régions et du manque d’accès aux soins des personnes atteintes d’épilepsie, le traitement antiépileptique est très lacunaire, les crises mal maîtrisées et les complications qu’elles causent représentent un fardeau considérable. De récentes enquêtes communautaires mettent en évidence la nécessité de stratégies de gestion de l’épilepsie adaptées aux villages isolés où l’onchocercose est endémique, dans le but d’améliorer la santé des personnes épileptiques.
Discussion : Les personnes atteintes d’épilepsie peuvent être mieux prises en charge dans les zones d’onchocercose endémique si le traitement de leur maladie est décentralisé. Des méthodes simplifiées de diagnostic et de traitement de l’épilepsie peuvent être employées par des non-médecins, sous la supervision de médecins ou de spécialistes. Afin de combler les lacunes du traitement, la fourniture régulière de médicaments antiépileptiques subventionnés, adaptés à différents types d’épilepsie associée à l’onchocercose, devrait être mise en place. L’établissement d’un système de surveillance communautaire de l’épilepsie permettra un diagnostic et un traitement précoces des personnes épileptiques, évitant ainsi les complications. Des programmes de sensibilisation sur l’épilepsie doivent être élaborés afin de réduire l’ostracisme et de faciliter la réinsertion sociale des personnes atteintes d’épilepsie. Enfin, renforcer les programmes d’élimination de l’onchocercose en optimisant le traitement à l’ivermectine sous directives communautaires (TIDC) et envisager d’autres stratégies de traitements pourrait réduire l’incidence de l’épilepsie.
Conclusions : Une approche communautaire, avec transfert des tâches de traitement de l’épilepsie de spécialistes vers des professionnels de la santé non-médecins, réduira la mortalité associée à l’épilepsie. Un plaidoyer et une collaboration accrus avec les différentes prenantes sont nécessaire

pour établir un modèle durable et économique de traitement de l'épilepsie, qui améliorera de façon significative la qualité de vie des personnes épileptiques dans les régions d'onchocercose endémique.

Translated from English version into French by Marie Anne, through

TRANSLATORS WITHOUT BORDERS

Совместное лечение эпилепсии в районах эндемичных для развития онхоцеркоза: лекции, созданные на базе коллективных исследований.

Джозеф Нельсон Сив Фоджо, Марике Ц. Дж. Деккер, Рихард Идро, Мишель Мандро Ндахура, Пьер-Мари Прё, Альфред К. Ньямнши и Роберт Колбандерс.

Аннотация

Введение: Считается, что в эндемичных для онхоцеркоза регионах, существует высокая вероятность развития эпилепсии. Ограниченные возможности лечения заболевания и отсутствие у больных доступа к медицинским учреждениям приводят к перерывам в лечении, к сокращению предупреждения случаев эпилептических припадков и повышению вероятности возникновения осложнений. Недавние исследования, проведённые среди местного населения, подчеркивают необходимость установления мер с целью снижения количества эндемичных для развития онхоцеркоза районов.

Содержание исследования: В случае децентрализации системы медицинской помощи больным эпилепсией, возможно улучшение ситуации в эндемичных для развития онхоцеркоза районах. Система рациональных подходов к диагностике и лечению заболевания эпилепсией может применяться техническим персоналом под руководством терапевтов и специалистов. Для максимального сокращения перерывов в лечении необходимо постоянное снабжение местности анти-эпилептическими препаратами, позволяющими бороться с различными видами эпилепсии на фоне онхоцеркоза. При создании местной системы мониторинга заболевания можно обеспечить раннюю диагностику и практиковать лечение пациентов, а также снизить риск возникновения осложнений. В целях облегчения процесса социальной реабилитации больных, следует проводить общественно-просветительские программы. В итоге, усиление программ по предупреждению онхоцеркоза с помощью инвермектина и других способов лечения способствует снижению риска возникновения заболевания.

Выводы: Данный подход к лечению эпилепсии, основанный на возможности лечения пациентов не врачами, а немедицинским персоналом, позволит в будущем снизить уровень смертности от эпилепсии. Активное содействие и помощь со стороны благотворительных организаций необходимы для создания устойчивой и материально-эффективной модели борьбы с эпилепсией. Тогда, качество жизни пациентов в районах эндемичных для развития онхоцеркоза значительно улучшится.

Translated from English version into Russian by Elena Chavykina, through

La atención integral para la epilepsia en zonas con oncocercosis endémica: lecciones aprendidas a partir de estudios de base comunitaria

Joseph Nelson Siewe Fodjo, Marieke C. J. Dekker, Richard Idro, Michel Mandro Ndahura, Pierre-Marie Preux, Alfred K. Njamnshi y Robert Colebunders

Resumen

Introducción: Las regiones donde la oncocercosis es endémica presentan una alta prevalencia de epilepsia. En estas zonas de bajos recursos, las personas epilépticas (PE) no disponen de acceso a asistencia sanitaria adecuada, lo que da lugar a una profunda brecha de tratamiento antiepiléptico, un control deficiente de las convulsiones y una gran carga de complicaciones relacionadas con las convulsiones. En estudios de base comunitaria recientes se pone de relieve la necesidad de diseñar y adoptar estrategias adecuadas para el diagnóstico y el tratamiento de la epilepsia en poblaciones remotas con oncocercosis endémica con el objetivo de asegurar mejores resultados sanitarios para las PE.

Texto principal: La descentralización de la asistencia para la epilepsia puede mejorar la atención sanitaria a PE en zonas con oncocercosis endémica. Por ejemplo, el personal no médico, bajo la supervisión de médicos o especialistas, podría utilizar protocolos simplificados de diagnóstico y tratamiento de la epilepsia. Para reducir la brecha de tratamiento, se ha de garantizar un suministro constante de fármacos antiepilépticos (FAE) subvencionados y adecuados para distintos tipos de epilepsia relacionada con la oncocercosis. Establecer sistemas de base comunitaria para la vigilancia de la epilepsia permitirá diagnosticar precozmente y tratar a PE y, por consiguiente, prevenir complicaciones relacionadas. Asimismo, han de llevarse a cabo programas comunitarios de concienciación sobre la epilepsia para disminuir el estigma asociado y facilitar la reinserción social de las PE. Por último, podría reducirse la incidencia de la epilepsia a través del refuerzo de los programas de eliminación de la oncocercosis mediante la optimización del tratamiento con ivermectina dirigido por la comunidad (CDTI, por sus siglas en inglés) y la puesta en marcha de estrategias alternativas de tratamiento.

Conclusiones: Adoptar un enfoque comunitario en el que el personal sanitario no médico pase a realizar las tareas de cuidado de la epilepsia desempeñadas hasta entonces por especialistas reducirá la morbilidad relacionada con la epilepsia. Es necesario aumentar las actividades de promoción y la colaboración con diversos agentes para establecer un modelo de asistencia a pacientes con epilepsia crónica que sea sostenible y rentable y con el que mejore considerablemente la calidad de vida de las PE en zonas con oncocercosis endémica.

Translated from English version into Spanish by Santiago Adrián Chambó, through

TRANSLATORS
WITHOUT BORDERS
