# Supplementary material for: Comprehensive management of epilepsy in onchocerciasis-endemic areas: lessons learnt from community-based surveys
Source: Infect Dis Poverty. 2019 Feb 10;8:11. doi: 10.1186/s40249-019-0523-y (PMC6368958; doi:10.1186/s40249-019-0523-y)
Supplement: Supplementary file 2 — Main components of a community-based epilepsy program. (DOCX 14 kb) [file 40249_2019_523_MOESM2_ESM.docx]

**Supplementary material 1:** Main components of a community-based epilepsy program

| **Aspect** | **Description** | **Activities** | **Parameters to monitor** |
| --- | --- | --- | --- |
| Screening and diagnosis | Simplified tools to facilitate the detection of PWE | - Train community health workers on the 5 questions  - Train health personnel on epilepsy diagnosis | - Percentage of enrolled persons enrolled in the epilepsy clinic, who will be confirmed as PWE by visiting physicians or specialists |
| Epilepsy treatment | Simplified approaches to AED prescription and basic management of complications | - Train local health personnel on AED prescription and monitoring treatment outcomes  - Training on management of complications | - Percentage of AED prescriptions and management plans judged as optimal when reviewed by visiting physicians or specialists |
| Epilepsy clinic | A health unit established within a healthcare facility, with personnel trained in basic epilepsy care including diagnosis, treatment and follow-up. | - Consultations, follow-up of PWE (monthly or otherwise, depending on seizure frequency)  - Regular supply of AED  - Supervision of Community Health Workers (CHW) | - Number of PWE registered and treated  - Frequency of seizures  - Number of complications, adverse effects or deaths amongst PWE  - Number of active CHW |
| Community Health Workers (CHW) | Proximity follow-up for PWE; serve as focal points for epilepsy care in the community. CHW are trained to counsel families and refer suspected PWE to the epilepsy clinic. | **-** Home visits to PWE  - Monitor seizure frequency, AED use and adverse effects  - Community sensitization  - First aid in case of seizures  - Refer suspected cases of epilepsy  - Epilepsy surveillance | - Number of home visits  - Number of educative talks delivered to community  - Number of new PWE referred  - Number of epilepsy-related events reported to the clinic |
| Community awareness program | Strategies to educate the population, stakeholders, health workers and teachers about epilepsy to reduce stigma | - Meetings with target audiences (school, market, church, authorities, communities, women associations) | - Number of people reached  - Behavioural change in the population |
| Onchocerciasis elimination  program | Ideally, a bi-annual CDTI program to reduce OAE and alternative treatment strategies if applicable. | - Community sensitization about the importance of ivermectin use.  - Ensure ivermectin use by all PWE. | - CDTI coverage in the village  - Epilepsy yearly incidence |
| Social rehabilitation program for PWE | Program to assist PWE to resume school and/or work, and better integrate the society | - Build skills in PWE via training sessions  - Assist school enrolment of PWE  - Establish associations for PWE | - Number of children with epilepsy who resume school or succeed professionally  - Major changes in quality of life (e.g. self-dependence, fulfilment, relationships/marriage) |
| Sustainability plan | Ensuring that the epilepsy program gets established | - Advocacy meetings with the elites, local authorities, communities, government  - Collaboration with social organizations | - Number of stakeholders contacted and collaborations initiated  - Number of PWE Associations  - Community involvement in epilepsy care |
