# Supplementary material for: Comprehensive management of epilepsy in onchocerciasis-endemic areas: lessons learnt from community-based surveys
Source: Infect Dis Poverty. 2019 Feb 10;8:11. doi: 10.1186/s40249-019-0523-y (PMC6368958; doi:10.1186/s40249-019-0523-y)
Supplement: Supplementary file 3 — Forms for epilepsy diagnosis and follow-up. (DOCX 80 kb) [file 40249_2019_523_MOESM3_ESM.docx]

**APPENDIX 1:**

**NEW PATIENT FORM (To be kept in patient file at the clinic)**

**Name of health facility:___________________________________ Date:_____________________**

| 1. **SOCIO-DEMOGRAPHIC INFORMATION** | | | | |
| --- | --- | --- | --- | --- |
| Name: | | | |  |
| Sex: | | | |  |
| Age: | | | |  |
| Level of Education: | | | |  |
| Profession: | | | |  |
| Address and Phone number: | | | |  |
| Name and telephone number of corresponding CHW: | | | |  |
| 1. **SEIZURE-RELATED HISTORY** | | | | |
| Age at First seizure without fever: | |  | | |
| Date of last seizure without fever: | |  | | |
| Description of seizure:   - Brief loss of consciousness - Jerky movements of limb(s) - Loss of urine - Drooling (saliva/foam) - Brief abnormal sensations - Head nodding movements - Others: | | Yes No if Yes, describe:______________________  Yes No if Yes, describe:______________________  Yes No if Yes, describe:______________________  Yes No if Yes, describe:______________________  Yes No if Yes: Hearing? Vision? Smell?  Yes No if Yes, describe:______________________ | | |
| Seizure frequency (for each type)  (per day/month/year): | |  | | |
| Past Antiepileptic treatment: | | *(name, dosage, daily intake)* | | |
| Present antiepileptic treatment: | | *(name, dosage, daily intake)* | | |
| Birth conditions | | Normal Difficult Caesarean section  Describe if abnormal:____________________________________ | | |
| Convulsion with fever? | | Yes No if Yes, at what age?:__________________ | | |
| Previous neurological condition? | | None Cerebral malaria Encephalitis/meningitis  Head trauma Other:_________________________ | | |
| Family member(s) with epilepsy? | | Yes No if Yes, precise:______________________ | | |
| Other relevant medical history | |  | | |
| 1. **ONCHOCERCIASIS-RELATED HISTORY** | | | | |
| Previous ivermectin intake: | | | | *(number of times, last year taken)* |
| Frequent itching: | | | | Yes No |
| Blurred vision: | | | | Yes No |
| 1. **PHYSICAL EXAMINATION** | | | | |
| Parameters: | | | **Temperature:**_____°C **Weight:** _____Kg **Height**:_____m  **Blood Pressure**:________mmHg **Other**:________________ | |
| General state: | | | Normal Moderately altered Poor  If abnormal, describe:___________________________________ | |
| Examination:   - Traumatic lesions - Burns or scars of burns - Leopard skin - Other skin abnormalities - Onchocercal nodules - Neurological examination: | | | Yes No if Yes, describe:______________________  Yes No if Yes, describe:______________________  Yes No if Yes, describe:______________________  Yes No if Yes, describe:______________________  Yes No if Yes, describe:______________________  Normal Mental disorder Muscle weakness  If abnormal, describe:___________________________________ | |
| Other relevant physical findings | | |  | |
| 1. **QUALITY OF LIFE** | | | | |
| Ability to perform at school or work at a job? | Normal Moderately affected Unable  If abnormal, describe:___________________________________ | | | |
| Ability to work at home or in the farm? | Normal Moderately affected Unable  If abnormal, describe:___________________________________ | | | |
| How would you rate your quality of life? | Very poor Poor Neither poor nor good  Good Very good  Remarks:______________________________________________ | | | |
| Other relevant findings |  | | | |
| 1. **PARACLINICAL FINDINGS** | | | | |
| Skin snip | | | Positive Negative  If positive, microfilarial load:_____________________________ | |
| OV16 serology | | | Positive Negative | |
| Malaria test | | | Positive Negative | |
| EEG | | |  | |
| Others | | |  | |

**ANY OTHER RELEVANT INFORMATION: _____________________________________________________**

**DIAGNOSIS:___________________________________________________________________________**

**TREATMENT INITIATED: _________________________________________________________________**

**EPILEPSY EDUCATION DONE?**  Yes No **PATIENT REFERRED?**  Yes No

**NEXT APPOINTMENT:**

**Name and Signature of Health Personnel: __________________________________**

**APPENDIX 2: Date:_____________________**

**MONTHLY FOLLOW UP FORM (To be archived in patient file at the clinic)**

| 1. **PATIENT IDENTIFICATION** | |
| --- | --- |
| Name: |  |
| Sex: |  |
| Age: |  |
| Address and Phone number: |  |
| Name / phone number of CHW: |  |
| Monthly expenses due to epilepsy: | *(AED, transport, Lost days of work*) |
| Date of last medical visit |  |
| 1. **SEIZURE-RELATED INFORMATION** | |
| Number of seizures since the last appointment: |  |
| Description of seizures: |  |
| Seizure frequency (for each type) | *(per day/month/year)* |
| Increase or decrease of seizures? |  |
| 1. **TREATMENT-RELATED INFORMATION** | |
| Current antiepileptic treatment | *(name, dosage, daily intake)* |
| Adherence to treatment? | Yes No (Reason:___________________________________)  If No, how many days missed? (count remaining AED):_________ |
| Any other treatment in addition to the prescription? | Yes No if Yes, specify:________________________ |
| 1. **QUALITY OF LIFE** | |
| Ability to perform at school or work at a job? | Normal Moderately affected Unable  If abnormal, describe:___________________________________ |
| Ability to work at home or in the farm? | Normal Moderately affected Unable  If abnormal, describe:___________________________________ |
| How would you compare your current quality of life with your state during the last appointment? | Much better Better Same  Worse Much worse  Remarks:_____________________________________________ |
| Other relevant findings |  |
| 1. **PHYSICAL EXAMINATION** | |
| New traumatic lesions/burns? | Yes No  If Yes, describe:________________________________________ |
| Other relevant findings: |  |
| 1. **CONCLUSION AND MANAGEMENT** | |
| Patient Evolution | Improvement Degradation Same  If other diagnosis, precise:_______________________________ |
| Treatment | Same Modified  Precise drug/dosage:___________________________________ |
| Next appointment | Date:_____________ Referred (precise):______________ |

**Name and signature of Health Personnel: _______________________________________________**

**APPENDIX 3:**

**MONTHLY COMMUNITY HEALTH WORKER FORM (Handed to the clinic at the end of each month)**

**Name of patient:_________________________________________ Month: ______________Year_____**

**Current treatment regimen: ______________________________________________________________**

| 1. **ACTIONS CARRIED OUT BY CHW FOR THE MONTH** | | | | | |
| --- | --- | --- | --- | --- | --- |
| Number of home visits to the family | | | |  | |
| Number of health talks delivered to the family | | | |  | |
| 1. **SEIZURE FREQUENCY AND AED INTAKE** | | | | | |
| **Date** | **Number of seizures** | **Number of AED received** | **Number of AED remaining** | | **Remarks** |
| 1 |  |  |  | |  |
| 2 |  |  |  | |  |
| 3 |  |  |  | |  |
| 4 |  |  |  | |  |
| 5 |  |  |  | |  |
| 6 |  |  |  | |  |
| 7 |  |  |  | |  |
| 8 |  |  |  | |  |
| 9 |  |  |  | |  |
| 10 |  |  |  | |  |
| 11 |  |  |  | |  |
| 12 |  |  |  | |  |
| 13 |  |  |  | |  |
| 14 |  |  |  | |  |
| 15 |  |  |  | |  |
| 16 |  |  |  | |  |
| 17 |  |  |  | |  |
| 18 |  |  |  | |  |
| 19 |  |  |  | |  |
| 20 |  |  |  | |  |
| 21 |  |  |  | |  |
| 22 |  |  |  | |  |
| 23 |  |  |  | |  |
| 24 |  |  |  | |  |
| 25 |  |  |  | |  |
| 26 |  |  |  | |  |
| 27 |  |  |  | |  |
| 28 |  |  |  | |  |
| 29 |  |  |  | |  |
| 30 |  |  |  | |  |
| 31 |  |  |  | |  |

**Name and signature of CHW: __________________________________________**

**APPENDIX 4:**

**MONTHLY EPILEPSY CLINIC REPORT (To be archived by clinic head)**

**Health Facility_____________________________ District: __________________Month/Year:________**

| 1. **PWE CONSULTED AT THE EPILEPSY CLINIC** | | | | | | | | | | |
| --- | --- | --- | --- | --- | --- | --- | --- | --- | --- | --- |
| **AGE GROUP** | | | **CASES SEEN** | | | | | | | **TOTAL** |
|  |  |  | **Male** | | | **Female** | | | |  |
|  |  |  | **New cases** | **Follow-up cases** | | **New cases** | | **Follow-up cases** | |  |
| < 5years | | |  |  | |  | |  | |  |
| 5 – 15 years | | |  |  | |  | |  | |  |
| 16 – 30 years | | |  |  | |  | |  | |  |
| > 30 years | | |  |  | |  | |  | |  |
| **TOTAL** | | | **A=** | **B=** | | **C=** | | **D=** | | **E=** |
| Total Number of epilepsy cases seen (E):___________  Number of new cases of epilepsy (A+C):___________  Number of Follow-up cases of epilepsy (B+D):___________  Number of new PWE with positive skin snip or OV16 seropositivity:__________  Number of PWE referred to specialists:___________  Number of deaths among PWE:_________  Other remarks: | | | | | | | | | | |
| 1. **CLINIC DETAILS** | | | | | | | | | | |
| Clinic staff (number/qualifications) | |  | | | | | | | | |
| Number of active CHW | |  | | | | | | | | |
| Number of support group sessions | | *(precise days and time)* | | | | | | | | |
| Visits from specialist | | Yes No if Yes, specify:_______________________ | | | | | | | | |
| 1. **ANTIEPILEPTIC DRUGS** | | | | | | | | | | |
| **Drug Name** | **Continually available? (Y/N)** | | **If No, Number of days out of stock** | | **Stock at start of month** | | **Stock at end of month** | | **Remarks** | |
| Phenobarbital  Dosage: |  | |  | |  | |  | |  | |
| Carbamazepine  Dosage: |  | |  | |  | |  | |  | |
| Phenytoine  Dosage: |  | |  | |  | |  | |  | |
| Valproate  Dosage: |  | |  | |  | |  | |  | |
| Other: |  | |  | |  | |  | |  | |
| Monthly cost price of AED | | |  | | | | | | | |
| Monthly selling price of AED | | |  | | | | | | | |
| Additional income for AED purchase | | | *(amount and source)* | | | | | | | |
| Number of AED suppliers | | |  | | | | | | | |
| Method of distribution of AED | | | Home delivery by CHW PWE come to the clinic  PWE buy at dispatch points Others: | | | | | | | |
| Difficulties or remarks on AED | | |  | | | | | | | |

| 1. **EXTERNAL CLINIC ACTIVITIES** | |
| --- | --- |
| Number of epilepsy health talks: ____  (in schools, market, church, etc) | *(precise venue and estimated audience)* |
| CHW activities | Home visits (Total number of home visits:___________)  New PWE referred to clinic (Total number: _________)  Epilepsy education (Number of families educated: _____)  Others: |
| Number of epilepsy outreach to remote areas by mobile health team | *(precise venue and estimated audience)* |
| New collaborations | Local authorities (specify:__________________________)  Traditional healers (Number:___________)  Public stakeholders (Specify: ________________________)  Private stakeholders (specify: _______________________)  Others: |

| 1. **GENERAL REMARKS FOR THE MONTH** |
| --- |
|  |

| 1. **PLANS FOR THE NEXT MONTH** | |
| --- | --- |
| Target number of health talks and venues |  |
| Target epilepsy clinic attendance |  |
| Target number of new PWE to enrol |  |
| Target number of homes to visit |  |
| Mobile epilepsy outreach to be done |  |
| New collaborations to be initiated |  |
| Other specific objectives: |  |

**Date:______________**

**Name, signature and official stamp of Clinic Head:___________________________________________**
